# Respiratory Function Tolerance of Rats with Vaying Degrees of Thoracic Volume Reduction

**DOI:** 10.1111/os.13630

**Published:** 2023-03-01

**Authors:** Jiuxu Deng, Xiao‐Kun Chen, Fu‐Zheng Guo, Wei Huang, Feng‐Xue Zhu, Tian‐Bing Wang, Bao‐Guo Jiang

**Affiliations:** ^1^ National Center for Trauma Medicine, Trauma Medicine Center, Ministry of Education Key Laboratory of Trauma and Neuroregeneration Peking University People's Hospital Beijing China

**Keywords:** Animal Model, Respiratory Function, Rib Fractures, Thoracic Volume, Three‐Dimensional Imaging

## Abstract

**Objective:**

To compare the effects of respiratory function on different degrees of reduced thoracic volume and evaluate the tolerance of rats with reduced thoracic volume, and to assess the feasibility of thoracic volume as a measure of the severity of rib fractures.

**Methods:**

A total of 24 10‐week‐old female Sprague–Dawley (SD) rats were randomly divided into four groups (n = 6 in each group) according to the displacement degree of bilateral rib fractures (2, 4, 6, and 8 mm). The respiratory function of the rats（Tidal volume, Inspiration time, Expiration time, Breath rate, Minute volume, Peak inspiration flow) measured *via* whole‐body barometric plethysmography before and after operation for 14 consecutive days. Respiratory function parameters of each group were analyzed. Chest CT scans were performed before and 14 days after operation, after that we reconstructed three‐dimensional of the thoracic and lung and measured their volumes by computer software. We calculated the percentage of thoracic and lung volume reduction after operation.

**Results:**

At the 14th day after the operation, the decline of thoracic volume rates of in the 2, 4, 6, and 8 mm groups were 5.20%, 9.01%, 16.67%, and 20.74%, respectively. The 8 mm group showed a significant reduction in lung volume. The postoperative tidal volumes were lower in each of the groups than the baseline values before the operation. The tidal volume of the 2 mm group gradually recovered after the operation and returned to a normal level (1.54 ± 0.07 mL) at 14th day after the operation. The tidal volume of the 4, 6, and 8 mm groups recovered gradually after the operation, but did not return to baseline level at the 14th day. In particular, the tidal volume of the 8 mm group was significantly lower than that of the other groups during the 14 days (1.23 ± 0.12 mL, *p* < 0.05). There were no significant changes in the inspiratory and expiratory times, peak inspiratory and expiratory flows, respiratory rate, and minute ventilation during the 14 days after the operation in each group.

**Conclusions:**

Displaced rib fractures lead to thoracic collapse and reduced thoracic volume, which can affect tidal volume in rats. The greater the decrease of thoracic volume, the more obvious the decrease of early tidal volume. The thoracic volume can be used as an objective parameter to evaluate the severity of multiple rib fractures. Early operation to restore thoracic volume may improve early respiratory function. Decreased thoracic volume affected respiratory function and can be compensated and recovered in the long term.

## Graphical abstract

In this study, we made four groups of animal models of multiple rib fractures in rats to compare the effects of different degrees of reduced thoracic volume on respiratory function tolerance.

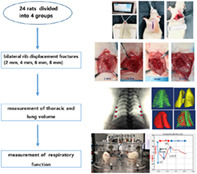

## Introduction

Rib fractures are common chest trauma, accounting for approximately 21% of all patients with blunt chest trauma.^1^ Severe rib fractures, such as flail chest and multiple rib fractures, have a mortality rate higher than 20%.^2^, ^3^, ^4^ Severe rib fractures often collapse into the thorax, leading to decreased respiratory activity and affecting thoracic volume, which subsequently affects cardiopulmonary function, resulting in decreased chest and lung compliance and impaired respiratory function. Previous studies have suggested that thoracic volume will be significantly reduced if severe chest collapse is not corrected, and the reduction in thoracic volume will directly affect lung volume and recruitment, ultimately affecting long‐term lung ventilation to varying degrees.^5^, ^6^

The published literature and expert consensus have focused on the treatment of flail chest or rib fractures alone, and there is a lack of sufficient research on the need for surgical treatment for severe non‐flail chest or combined injuries (such as severe cranial injury).^7^, ^8^ There are two main reasons: one is the lack of a reliable way to evaluate the severity of multiple rib fractures; and the other is that the relationship between multiple rib fractures and respiratory function is not that clear. In the past, clinicians evaluated the severity of multiple rib fractures in patients by the number of rib fractures and respiratory function parameters. Some studies have shown that the more fractures the patients have, the higher the mortality rate and the incidence of pneumonia will be.^9^, ^10^, ^11^ However, these two methods of evaluating the severity of multiple rib fractures have some limitations: (i) the situation of multiple rib fracture is complex, the prognosis of patients is different with different location and displacement of fractures. Usually, we choose conservative treatment if the fractures at the 1st–3rd ribs and 10th–12th ribs or fractures with no obvious displacement, which have little influence on respiratory function; (ii) many other factors besides the rib fractures affect respiratory function such as pulmonary contusion and hemopneumothorax. So, it is not accurate to use respiratory function to evaluate the severity of multiple rib fractures. Multiple rib fractures with pulmonary contusion should be carefully considered for SSRF, and the effect of rib fractures on respiratory function should be accurately evaluated. Displaced rib fractures directly affect thoracic volume, so the study of the relationship between thoracic volume reduction and respiratory function changes after multiple rib fractures is of great significance in clinical severity evaluation and guidance of treatment decisions. There are many complications affecting respiratory function in patients with multiple rib fractures, while the animal model of isolated multiple rib fractures can avoid the interference of other factors.

The purpose of the present study was: (i) to establish isolated multiple rib fracture rat models and evaluate the validity and stability of the models; (ii) to compare the effects of respiratory function on different degrees of reduced thoracic volume and evaluate the tolerance of rats with reduced thoracic volume; (iii) to assess the feasibility of thoracic volume as a measure of the severity of rib fractures.

## Methods

### Experimental Animals and Grouping

Twenty‐four 10‐week‐old, clean grade, female SD rats (The Animal Experiment Center of Peking University People's Hospital, Beijing, China), weighing 220–240 g, were selected for this study. This experiment strictly complied with the Chinese Guidelines for Welfare and Use of Experimental Animals and received approval from the Ethics Committee of Peking University People's Hospital (approval number was 2020PHB103‐01). All experimental procedures were performed according to best practices for reducing the number of experimental animals and animal suffering. The animals were randomly divided into four groups (n = 6 in each group) and received surgeries with different degrees of bilateral rib displacement (2, 4, 6, and 8 mm).

### Establishment of Multiple Rib Fracture Models with Different Degrees of Displacement

**Figure 1:**
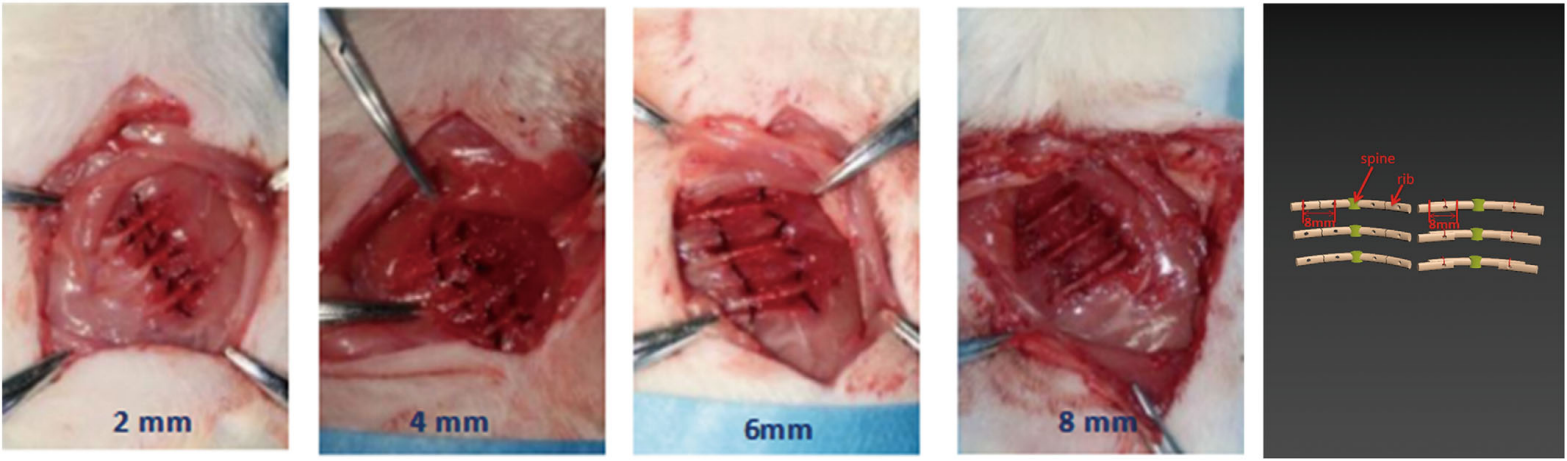
Making the multiple rib fractures models of 2, 4, 6, 8 mm displacement degree

After the animals were weighed, anesthesia was induced using 5% isoflurane (flow 1 L/min) and maintained through inhalation of 1%–3% isoflurane. The rats were fixed on the microsurgery table in the supine position, and 1% bupivacaine was injected subcutaneously at the operation site to prevent tension pneumothorax due to a surgical accident or rib fracture during the preparation of the animal model. To ensure safety during the operation, noninvasive tracheal intubation was used to connect the ventilator to the anesthesia machine. The rats' bilateral chest skin was prepared and sterilized with routine disinfection cloth. The skin, superficial and deep fascia, and muscle tissue were cut through the left axillary midline ribs (ribs 5th‐7th) in turn, and the ribs were carefully exposed and separated. During the operation, the intercostal and other respiratory muscles were carefully protected. After the ribs were exposed, a skull bone drill was used to drill a hole (diameter = 0.5 mm) at 2 mm between the rib diaphysis, and the ribs were transected at the midpoint of the two holes. A 4–0 suture line was passed through the hole, the distal and proximal segments were fixed, and the 2 mm displacement model of a rib fracture was complete. Physiological saline was used to wash the wound, and the incision was closed layer by layer. The 2 mm displacement model of a right rib 5th–7th fractures were created first. Bilateral multiple rib fracture displacement models of 4, 6, and 8 mm were then constructed according to the same procedure (Figure 1).

### Micro‐CT Scan

Fourteen days after the operation, the rats were anesthetized with 1% pentobarbital sodium (4 mL/kg) by intraperitoneal injection. After the anesthetic took effect, a chest micro‐CT image was taken (GE Healthcare, USA) with the following scanning parameters: working voltage (80 kV), machine current (450 μA), spatial resolution (0.046 mm), exposure time (400 ms), and scanning time (24 min). The target area was analyzed and reconstructed using Microview 3D imaging software.

### Reconstruction of the 3D Thoracic Model

The rat chest CT data (in DICOM format) were imported into Materialize's Interactive Medical Image Control System (MIMICS, version 14Materialise, Leuven, Belgium), and we entered the coronal, sagittal, and cross‐sectional operation window parameters to extract the contours of the ribs, lung tissue, and chest contents using density threshold segmentation. We then used this information to generate a 3D model. The volume of the reconstructed 3D model was quantified by surveying and mapping functions within the MIMICS software.

### Noninvasive Lung Function Test

**Figure 2:**
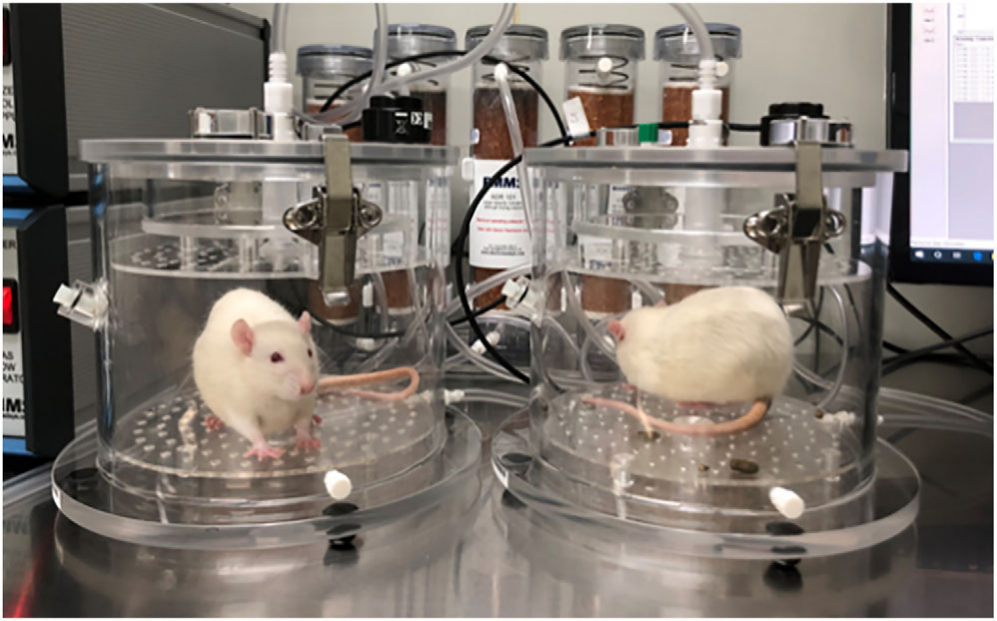
Testing two Sprague–Dawley (SD) rats with the whole‐body barometric plethysmography (WBBP) body scanner. When the rats breathe, the gas pressure in the container changes, and the signal is collected by the instrument

Whole‐body barometric plethysmography (WBBP) is a non‐invasive and objective technique for measuring the respiratory function of animals that are completely awake with minimal restrictions. The rats were placed in a body scanner (EMMS, Hants, UK), and the respiratory function baseline data were collected before constructing the rat model. eDacq software (EMMS, Hants, UK) was used to digitally transform the collected signal. After the rats adapted to the test environment for 5–10 min, measurement results were recorded every 4 min (Figure 2).

**Figure 3:**
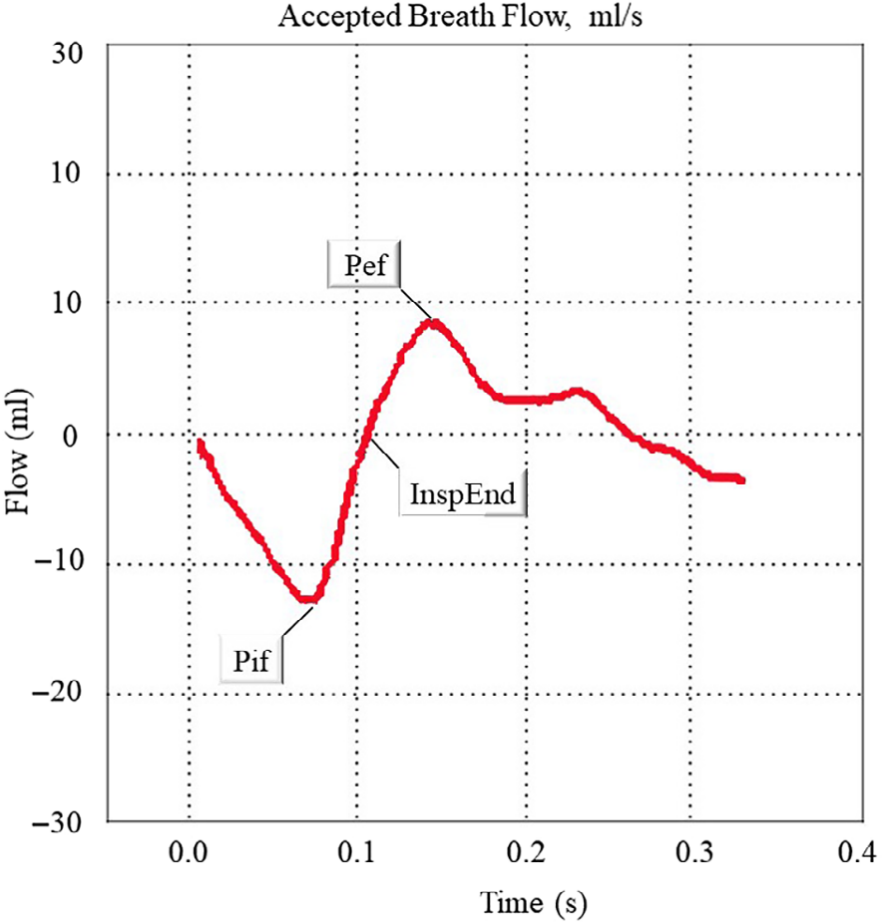
Schematic diagram of WBBP(The whole‐bodybarometric plethysmography) waveform of a respiratory cycle. Respiratory function parameters of rats can be obtained. Pif: Peak inspiratory flow; Pef: Peak expiration flow; InspEnd: End of Inspiration

The respiratory parameters selected for analysis were the following (Figure 3):

Inspiration time (Ti, s): The time elapsed during each breath cycle from inhale to exhale.

Expiration time (Te, s): The time elapsed during each breath cycle from exhale to inhale.

Tidal volume (TV, ml): The amount of air inhaled or exhaled at a time of calm breathing.

Peak inspiratory flow (PIF, ml/s): The maximum amount of gas inhaled per unit time.

Peak expiration flow (PEF, ml/s): The maximum amount of gas exhaled per unit time.

Breath rate (breaths/min): Breaths per minute.

Minute volume (MV, ml): The amount of air inhaled or exhaled from the lungs per minute.

All the above parameters indicate the ventilation function of the lung.

The eDacq software was used to automatically mark the start and end of each respiratory cycle. The respiratory cycle used in the study indicated that the difference between inhalation and expiration was within 20%, and the rats were in a relaxed, still state and were not breathing. The animals' respiratory parameters were tested after the operation for 14 consecutive days.

### Statistical Analysis

The average value of each index data measured by 4 min of each rat was taken as the variable value. All the statistical calculations were performed using SPSS 19.0 statistical software (IBM, Armonk, New York, USA), and the data were expressed as the mean ± SD, and two independent sample *t*‐tests were used to compare the data between the groups. *P* < 0.05 was considered statistically significant.

## Results

### General Observations after Surgery

All animals survived until the end of the study. One week after the operation, the redness and swelling of the incision disappeared, and the animals could eat and move normally.

### Respiratory Parameter Measurements

#### Tidal Volume

The tidal volume in each group was significantly lower after the operation compared to the preoperative baseline numbers (1.61 ± 0.08 mL); however, the tidal volume of the 2 mm group returned to the normal level by the 14th day (1.54 ± 0.07 mL). The tidal volumes of the 4 mm, 6 mm, and 8 mm displacement groups gradually recovered, but did not fully return to normal levels by the 14th day. In particular, the tidal volume of the 8 mm displacement group was still significantly lower than that of the other groups on the 14th day after the operation (1.23 ± 0.12 mL) (Table 1).

**Table 1:** Tidal volume (mL)

Group	2 mm group	4 mm group	6 mm group	8 mm group	*F* value	* *P* value
Preoperative	1.61 ± 0.08	1.61 ± 0.08	1.61 ± 0.08^d^	1.61 ± 0.08		
Postoperative						
Day 1	1.16 ± 0.16^d^	1.10 ± 0.06^d^	1.14 ± 0.06^d^	0.92 ± 0.11^b^ ^,^ ^d^	6.545	0.003*
Day 2	1.11 ± 0.05^d^	1.01 ± 0.06^a^ ^,^ ^d^	1.14 ± 0.11^b^ ^,^ ^d^	0.96 ± 0.08^a^ ^,^ ^c^ ^,^ ^d^	7.696	0.001*
Day 3	1.23 ± 0.08^d^	1.15 ± 0.10^d^	1.09 ± 0.10^d^	1.09 ± 0.13^d^	2.529	0.086
Day 4	1.23 ± 0.14^d^	1.21 ± 0.06^d^	1.18 ± 0.11^d^	1.03 ± 0.10^a^ ^,^ ^b^ ^,^ ^c^ ^,^ ^d^	4.329	0.017*
Day 5	1.24 ± 0.11^d^	1.20 ± 0.15^d^	1.08 ± 0.07^a^ ^,^ ^d^	1.09 ± 0.08^a^ ^,^ ^d^	3.261	0.043*
Day 6	1.34 ± 0.20^d^	1.17 ± 0.10^a^ ^,^ ^d^	1.23 ± 0.08^d^	1.1 ± 0.09^a^ ^,^ ^d^	3.806	0.026*
Day 7	1.36 ± 0.11^d^	1.23 ± 0.13^d^	1.22 ± 0.10^d^	1.20 ± 0.09^d^	2.774	0.068
Day 8	1.38 ± 0.11^d^	1.29 ± 0.07^d^	1.29 ± 0.10^d^	1.19 ± 0.09^a^ ^,^ ^d^	4.179	0.019*
Day 9	1.35 ± 0.09^d^	1.31 ± 0.08^d^	1.25 ± 0.09^d^	1.21 ± 0.08^d^	2.996	0.055
Day 10	1.43 ± 0.05^d^	1.34 ± 0.08^d^	1.27 ± 0.16^a^ ^,^ ^d^	1.19 ± 0.10^a^ ^,^ ^b^ ^,^ ^d^	5.268	0.008*
Day 11	1.43 ± 0.05^d^	1.37 ± 0.06^d^	1.30 ± 0.10^a^ ^,^ ^d^	1.20 ± 0.06^a^ ^,^ ^b^ ^,^ ^c^ ^,^ ^d^	11.915	0.000*
Day 12	1.50 ± 0.06^d^	1.36 ± 0.11^d^	1.31 ± 0.07^a^ ^,^ ^d^	1.15 ± 0.24^a^ ^,^ ^b^ ^,^ ^d^	6.333	0.003*
Day 13	1.51 ± 0.04^d^	1.31 ± 0.12^a^ ^,^ ^d^	1.27 ± 0.12^a^ ^,^ ^d^	1.25 ± 0.10^a^ ^,^ ^d^	8.499	0.001*
Day 14	1.54 ± 0.07	1.36 ± 0.09^a^ ^,^ ^d^	1.36 ± 0.07^a^ ^,^ ^d^	1.23 ± 0.12^a^ ^,^ ^b^ ^,^ ^c^ ^,^ ^d^	12.226	0.000*
*F* Value	19.624	29.760	27.187	33.330		
*P* value	0.000^#^	0.000^#^	0.000^#^	0.000^#^		

* comparison between groups.

^a^ Comparison with group 2 mm.

^b^ Comparison with group 4 mm.

^c^ Comparison with group 6 mm.

^d^ Comparison with the preoperative state.

^#^ Comparison within groups.

Significance: *P* < 0.05.

#### Inspiration Time

There was no statistically significant difference in the inspiratory times after surgery in any of the groups compared with the preoperative period (Table 2).

**Table 2:** Inspiraton time (s)

Group	2 mm group	4 mm group	6 mm group	8 mm group	*F* value	*P* value
Preoperative	0.17 ± 0.03	0.17 ± 0.03	0.17 ± 0.03	0.17 ± 0.03		
Postoperative						
Day 1	0.17 ± 0.02	0.14 ± 0.03	0.15 ± 0.02	0.19 ± 0.04^b^ ^,^ ^c^	3.577	0.032*
Day 2	0.21 ± 0.07	0.16 ± 0.05	0.17 ± 0.04	0.19 ± 0.08	0.784	0.517
Day 3	0.14 ± 0.02	0.14 ± 0.02	0.17 ± 0.06	0.18 ± 0.05	1.625	0.215
Day 4	0.17 ± 0.03	0.17 ± 0.06	0.16 ± 0.03	0.16 ± 0.01	0.059	0.981
Day 5	0.15 ± 0.02	0.15 ± 0.03	0.13 ± 0.02	0.19 ± 0.05^a^ ^,^ ^c^	4.106	0.020*
Day 6	0.19 ± 0.05	0.15 ± 0.01	0.17 ± 0.05	0.15 ± 0.04	1.108	0.369
Day 7	0.18 ± 0.05	0.18 ± 0.07	0.17 ± 0.03	0.16 ± 0.03	0.150	0.928
Day 8	0.16 ± 0.03	0.21 ± 0.07	0.18 ± 0.03	0.20 ± 0.06	0.963	0.430
Day 9	0.19 ± 0.05	0.18 ± 0.06	0.20 ± 0.07	0.17 ± 0.05	0.251	0.859
Day 10	0.19 ± 0.03	0.18 ± 0.04	0.21 ± 0.06	0.15 ± 0.03	2.110	0.131
Day 11	0.16 ± 0.03	0.17 ± 0.03	0.17 ± 0.05	0.19 ± 0.05	0.709	0.558
Day 12	0.18 ± 0.04	0.17 ± 0.02	0.16 ± 0.03	0.16 ± 0.05	0.735	0.543
Day 13	0.17 ± 0.02	0.19 ± 0.05	0.17 ± 0.03	0.15 ± 0.04	0.946	0.437
Day 14	0.18 ± 0.02	0.16 ± 0.05	0.15 ± 0.03	0.16 ± 0.03	0.773	0.523
*F* Value	1.467	1.137	1.612	0.964		
*P* value	0.139	0.338	0.090	0.495		

* Comparison between groups.

^a^ Comparison with group 2 mm.

^b^ Comparison with group 4 mm.

^c^ Comparison with group 6 mm.

Significance: P < 0.05.

#### Expiration Time

There was no statistically significant difference in the exhalation times after surgery in any of the groups compared with the preoperative period (Table 3).

**Table 3:** Expiration time (s)

Group	2 mm group	4 mm group	6 mm group	8 mm group	*F* value	*P* value
Preoperative	0.35 ± 0.09	0.35 ± 0.09	0.35 ± 0.09	0.35 ± 0.09		
Postoperative						
Day 1	0.33 ± 0.04	0.27 ± 0.05	0.31 ± 0.10	0.38 ± 0.10	2.194	0.120
Day 2	0.35 ± 0.08	0.30 ± 0.07	0.31 ± 0.11	0.28 ± 0.06	0.950	0.435
Day 3	0.30 ± 0.06	0.27 ± 0.07	0.31 ± 0.06	0.32 ± 0.06	0.870	0.473
Day 4	0.34 ± 0.09	0.31 ± 0.10	0.33 ± 0.07	0.32 ± 0.03	0.205	0.892
Day 5	0.31 ± 0.06	0.32 ± 0.07	0.25 ± 0.05	0.35 ± 0.08	2.262	0.112
Day 6	0.36 ± 0.07	0.31 ± 0.05	0.31 ± 0.09	0.29 ± 0.05	1.243	0.321
Day 7	0.32 ± 0.07	0.34 ± 0.10	0.34 ± 0.06	0.32 ± 0.08	0.115	0.950
Day 8	0.33 ± 0.06	0.38 ± 0.10	0.38 ± 0.07	0.38 ± 0.09	0.509	0.681
Day 9	0.35 ± 0.05	0.33 ± 0.08	0.33 ± 0.11	0.34 ± 0.10	0.074	0.973
Day 10	0.37 ± 0.06	0.32 ± 0.06	0.35 ± 0.05	0.28 ± 0.03^a^ ^,^ ^b^	3.154	0.047*
Day 11	0.33 ± 0.06	0.29 ± 0.08	0.33 ± 0.07	0.36 ± 0.07	1.198	0.336
Day 12	0.37 ± 0.05	0.36 ± 0.06	0.31 ± 0.08	0.27 ± 0.08	2.703	0.073
Day 13	0.32 ± 0.06	0.40 ± 0.13	0.35 ± 0.03	0.29 ± 0.06	1.816	0.177
Day 14	0.33 ± 0.04	0.32 ± 0.11	0.31 ± 0.09	0.32 ± 0.08	0.059	0.981
*F* Value	0.507	1.265	0.964	1.573		
*P* value	0.923	0.244	0.496	0.101		

* Comparison between groups.

^a^ comparison with group 2 mm.

^b^ comparison with group 6 mm.

Significance: *P* < 0.05.

#### Breath Rate

There was no statistically significant difference in the breath rates after surgery in any of the groups compared with the preoperative period (Table 4).

**Table 4:** Breath rate (breaths/min)

Group	2 mm group	4 mm group	6 mm group	8 mm group	*F* vValue	*P* value
Preoperative	133.13 ± 37.65	133.13 ± 37.65	133.13 ± 37.65	133.13 ± 37.65		
Postoperative						
Day 1	129.60 ± 19.93	164.52 ± 45.02	152.97 ± 42.98	111.32 ± 24.64	2.787	0.067
Day 2	118.75 ± 26.68	163.23 ± 48.97	153.28 ± 59.53	149 ± 46.69	0.998	0.414
Day 3	164.75 ± 39.08	173.55 ± 39.35	148.7 ± 53.74	136.48 ± 27.1	0.978	0.423
Day 4	143.27 ± 40.66	148.18 ± 53.35	143.75 ± 38.57	145.62 ± 21.31	0.019	0.996
Day 5	153.45 ± 34.67	144.00 ± 32.26	206.17 ± 46.01^a^ ^,^ ^b^	126.72 ± 27.77^c^	5.480	0.006*
Day 6	131.18 ± 36.13	149.15 ± 28.77	153.83 ± 61.18	169.65 ± 46.48	0.749	0.536
Day 7	143.28 ± 38.53	148.05 ± 56.12	134.08 ± 32.85	138.83 ± 28.2	0.132	0.940
Day 8	143.78 ± 43.96	117.77 ± 31.05	114.62 ± 21.59	116.42 ± 40.87	0.911	0.454
Day 9	123.88 ± 19.22	143.58 ± 55.24	129.65 ± 34.41	154.37 ± 56.49	0.583	0.633
Day 10	114.62 ± 18.59	135.03 ± 34.88	117.65 ± 33.51	159.95 ± 26.57	3.063	0.052
Day 11	146.28 ± 34.31	146.10 ± 41.09	146.30 ± 47.84	124.62 ± 33.45	0.447	0.722
Day 12	128.00 ± 21.49	127.75 ± 24.50	158.42 ± 46.41	175.03 ± 54.18	2.142	0.127
Day 13	131.73 ± 23.07	121.32 ± 47.43	124.08 ± 15.89	175.77 ± 54.82	2.566	0.083
Day 14	131.35 ± 11.19	153.45 ± 55.94	158.85 ± 49.20	148.83 ± 40.05	0.467	0.708
*F* Value	1.009	0.912	1.757	1.698		
*P* value	0.452	0.549	0.057	0.069		

* Comparison between groups.

^a^ Comparison with group 2 mm.

^b^ Comparison with group 4 mm.

^c^ Comparison with group 6 mm.

Significance: *P* < 0.05.

#### Minute Volume

There was no statistically significant difference in the minute volumes after surgery in any of the groups compared with the preoperative period (Table 5).

**Table 5:** Minute volume (ml)

Group	2 mm group	4 mm group	6 mm group	8 mm group	*F* value	*P* value
Preoperative	200.67 ± 51.63	200.67 ± 51.63	200.67 ± 51.63	200.67 ± 51.63		
Postoperative						
Day 1	141.68 ± 18.73^d^	172.7 ± 45.65	161.73 ± 41.00	100.87 ± 22.09^a^ ^,^ ^b^ ^,^ ^c^ ^,^ ^d^	5.219	0.008*
Day 2	126.82 ± 26.61^d^	149.15 ± 38.72	159.82 ± 39.11^d^	135.48 ± 34.36^d^	1.039	0.397
Day 3	186.93 ± 50.89	184.42 ± 37.43	150.67 ± 36.60^d^	144.60 ± 35.65^d^	1.780	0.183
Day 4	162.75 ± 28.33^d^	169.52 ± 53.53	156.87 ± 37.80^d^	137.58 ± 20.09^d^	0.824	0.496
Day 5	175.02 ± 35.55	163.02 ± 27.15	200.42 ± 41.82	131.42 ± 32.97^a^ ^,^ ^c^ ^,^ ^d^	4.072	0.021*
Day 6	162.70 ± 42.92	161.78 ± 27.18	174.17 ± 52.52	174.47 ± 41.78	0.165	0.919
Day 7	182.50 ± 52.13^d^	163.70 ± 49.80	154.12 ± 27.14^d^	158.10 ± 24.13^d^	0.581	0.634
Day 8	181.70 ± 35.95	143.40 ± 34.01	142.50 ± 31.06^d^	135.50 ± 49.09^d^	1.803	0.179
Day 9	160.08 ± 23.35^d^	171.02 ± 49.11	155.23 ± 39.73^d^	166.60 ± 53.63	0.157	0.924
Day 10	159.87 ± 26.13^d^	171.15 ± 32.40	143.17 ± 37.54^d^	180.53 ± 31.91	1.491	0.247
Day 11	193.20 ± 41.73	191.13 ± 49.76	177.22 ± 45.76	139.30 ± 31.79^d^	2.044	0.140
Day 12	174.43 ± 23.71	162.6 ± 24.31	190.15 ± 52.66	180.98 ± 30.64	0.662	0.585
Day 13	190.58 ± 33.14	149.67 ± 48.53	151.85 ± 23.13^d^	199.77 ± 60.85	2.099	0.133
Day 14	188.35 ± 14.09	197.27 ± 75.78	202.63 ± 65.09	172.50 ± 45.98	0.339	0.797
*F* value	2.151	1.291	1.935	3.806		
*P* value	0.016^#^	0.228	0.032^#^	0.000^#^		

* comparison between groups.

^a^ Comparison with group 2 mm.

^b^ Comparison with group 4 mm.

^c^ Comparison with group 6 mm.

^d^ Comparison with preoperative state.

^#^ Comparison within groups.

Significance: *P* < 0.05.

#### Peak Inspiration Flow

There was no statistically significant difference in the peak inspirations after surgery in any of the groups compared with the preoperative period (Table 6).

**Table 6:** Peak inspiration flow (mL/s)

Group	2 mm group	4 mm group	6 mm group	8 mm group	*F* value	*P* value
Preoperative	14.46 ± 2.29	14.46 ± 2.29	14.46 ± 2.29	14.46 ± 2.29		
Postoperative						
Day 1	10.40 ± 1.47^d^	11.56 ± 3.30^d^	11.85 ± 2.22^d^	7.09 ± 1.21^a^ ^,^ ^b^ ^,^ ^c^ ^,^ ^d^	5.865	0.005*
Day 2	9.16 ± 2.14^d^	10.55 ± 2.64^d^	10.84 ± 2.10^d^	8.77 ± 2.79^d^	1.049	0.393
Day 3	14.01 ± 2.87	12.87 ± 2.11	10.30 ± 2.82^d^	11.06 ± 4.82^d^	1.564	0.229
Day 4	11.71 ± 2.18^d^	11.83 ± 3.86^d^	11.54 ± 2.42^d^	9.93 ± 1.36^d^	0.697	0.565
Day 5	13.35 ± 2.33	11.94 ± 2.19^d^	14.01 ± 2.08	9.64 ± 2.51^a^ ^,^ ^c^ ^,^ ^d^	4.313	0.017*
Day 6	11.72 ± 3.32^d^	12.09 ± 1.65	11.63 ± 2.92^d^	12.46 ± 2.78	0.114	0.951
Day 7	12.50 ± 3.68	11.69 ± 3.70^d^	11.15 ± 1.35^d^	11.46 ± 2.02^d^	0.240	0.867
Day 8	13.23 ± 2.45	10.45 ± 2.75^d^	11.12 ± 2.44^d^	10.03 ± 3.47^d^	1.532	0.237
Day 9	11.65 ± 2.39^d^	12.02 ± 3.34	10.61 ± 3.03^d^	12.02 ± 3.21	0.295	0.829
Day 10	11.53 ± 2.18^d^	11.64 ± 2.27^d^	9.85 ± 3.17	12.68 ± 2.88	1.161	0.349
Day 11	14.03 ± 2.01	11.88 ± 2.69^d^	13.20 ± 3.84	10.20 ± 2.49^d^	2.077	0.135
Day 12	13.40 ± 3.02	12.19 ± 1.25	13.45 ± 2.87	12.27 ± 2.40	0.465	0.710
Day 13	13.17 ± 1.57	11.24 ± 2.00^d^	11.97 ± 2.18^d^	13.77 ± 2.66	1.718	0.196
Day 14	13.66 ± 1.37	14.13 ± 4.25	14.72 ± 3.23	12.69 ± 3.12	0.442	0.725
*F* Value	2.962	1.832	3.064	4.706		
*P* value	0.001^#^	0.045^#^	0.001^#^	0.000^#^		

* Comparison between groups.

^a^ Comparison with group 2 mm.

^b^ Comparison with group 4 mm.

^c^ Comparison with group 6 mm.

^d^ Comparison with preoperative state.

^#^ Comparison within groups.

Significance: *P* < 0.05.

### Changes in Thoracic Volume and Lung Volume

**Figure 4:**
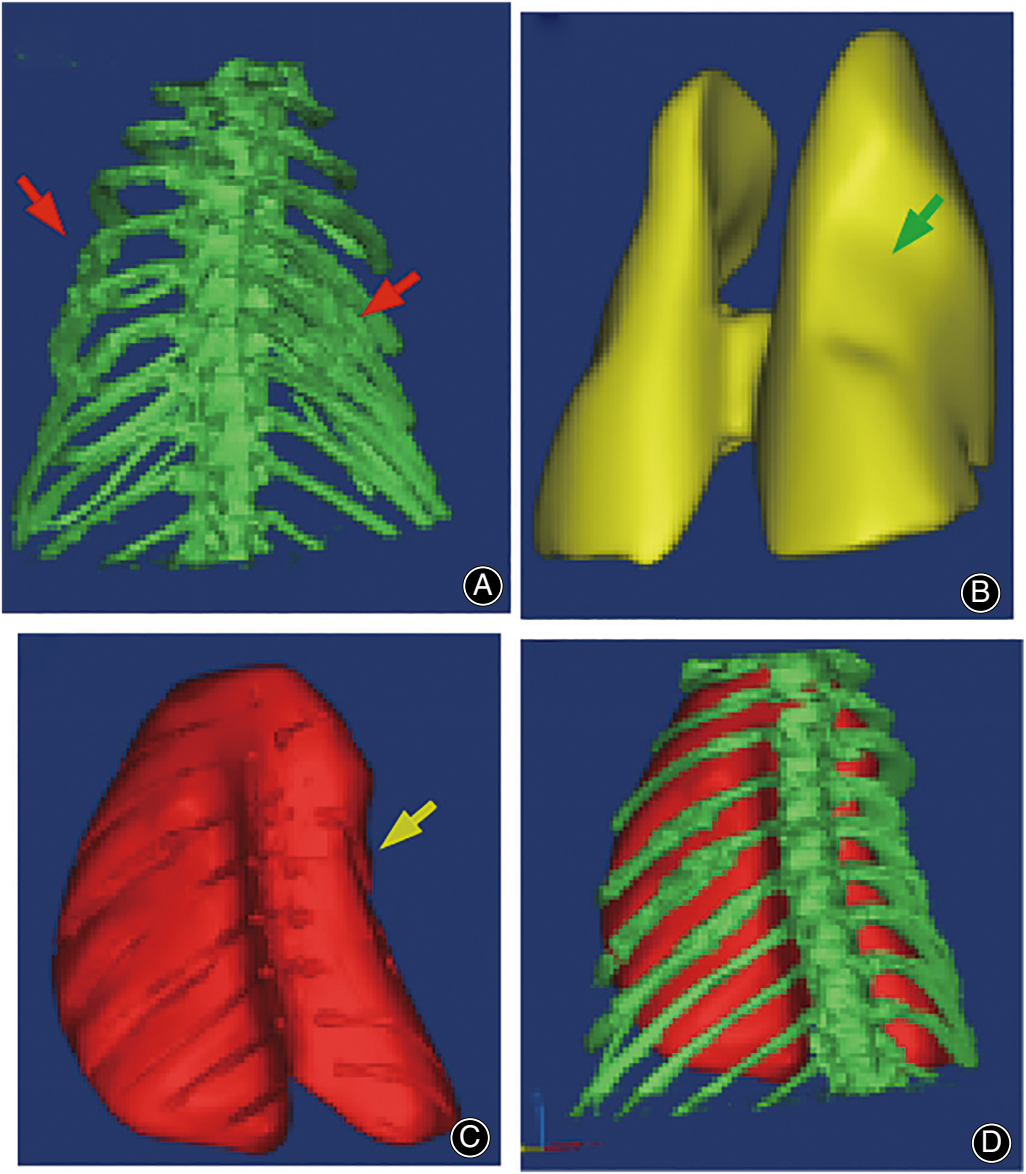
This figure shows an 8 mm group thoracic 3D reconstruction after 2 weeks of operation. (A) 3D reconstruction of the thoracic bony structure, and the porosis can be seen (red arrow). (B) 3D reconstruction of lung tissues, and the collapse of partial lung tissues can be seen (green arrow). (C) Reconstruction of the contents in the entire thorax, and the collapse of partial thorax can be seen (yellow arrow). (D) 3D reconstruction of the entire thorax

The micro‐CT analysis showed that, 14 days after the operations, each group experienced differing degrees of healing. After reconstruction of the 3D chest model, the thoracic volume and lung volume changes rate in the rats were calculated (Figure 4). Compared with the normal group, the thoracic volume change rates of the 2, 4, 6, and 8 mm displacement groups were 5.20%, 9.01%, 16.67%, and 20.74%, respectively, the lung volume change rates were 5.26%, 6.83%, 11.11%, and 14.77%, respectively (Table 7).

**Table 7:** Thoracic and lung volume change rate

Group (mm)	Thoracic volume (mm^3^)	Lung volume (mm^3^)	Thoracic volume change rate (%)	Lung volume change rate (%)
2	10811.9	5772.24	5.13%	5.26%
4	10379.25	5676.48	8.93%	6.83%
6	9497.68	5416.03	16.66%	11.11%
8	9032.13	5192.61	20.75%	14.77%
Normal	11396.88	6092.74	null	null

## Discussion

### Research Advantages of the Rat Model of Rib Fracture

Rib fractures are common in chest trauma, multiple rib fractures can significantly increase the incidence of pneumonia and patients' mortality.^9^ Studies have shown that the number of displaced fractures may be a strong predictor of pulmonary complications, and associated with higher mortality.^10^, ^11^ However, human has a large number of ribs, and the anatomical structure of ribs is complex, as each rib is different in length and shape. When rib fractures occur, there are various fracture sites and types, so it is controversial to judge the severity of multiple rib fracture based on the number of rib fractures. Most rib fractures occur in the lateral ribs (the 4th–9th rib). These ribs are slender, and when the chest is subjected to direct or indirect violence, the middle section of the ribcage becomes the central point of the stress, so the lateral ribs are prone to fracture.^12^ The thoracic volume reduced after rib fractures, and the degree of reduction is affected by fracture displacement and collapse, as well as the number of ribs affected and the location of the fracture.^13^ At present, the optimal treatment of multiple rib fractures is controversial therapy and many of the current theories are not supported by high‐level clinical evidence. As a result, the treatment of multiple rib fractures is frequently guided by expert consensus or clinical experience.^14^, ^15^ In clinical practice, however, patients with rib fractures usually have pulmonary contusions and severe pain, and these factors affect patients' respiratory function to varying degrees.^16^, ^17^ In addition, some patients may have experienced cardiopulmonary insufficiency in the past. All these factors work together to cause respiratory dysfunction in patients with chest trauma. However, the effect of reduced thoracic volume due to chest wall deformity caused by rib fractures on respiratory function remains unclear. We aimed to simulate a simple rat model with multiple rib fractures to explore the influence of a reduction in thoracic volume on respiratory function after multiple rib fracture displacement. In this study, the ribs were cut without puncturing the pleura. In contrast to rib fractures made by the hit model, we were able to avoid pulmonary contusions and blood pneumothorax during the creation of the rat models. In addition, we fixed the displaced ribs to avoid, as much as possible, respiratory function interference caused by pain.

### Effect of Reduced Thoracic Volume on Respiratory Function in Rats

This study showed that tidal volume was the most influenced parameter after the thoracic volume was reduced. The tidal volumes measured 2 weeks after the operation were significantly lower than the preoperative baselines, especially in the 8 mm group, where the thoracic volume was reduced by approximately 20%. The results of the thorax and lung tissue 3D reconstructions (Table 7) show that the thoracic and lung volume were reduced in each group. The 6 and 8 mm groups experienced a significant reduction in thoracic volume, while a reduction of the lung volume occurred in only the 8 mm group. The tidal volume of each group gradually returned to nearly normal within 2 weeks after the operation, but there was little change in the other indexes. We found that the decrease in tidal volume was due to restrictive ventilatory dysfunction caused by the reduced thoracic volume. The tidal volumes gradually returned to normal because the rats gradually became tolerant to the reduced thoracic volume and were able to compensate for the ventilation dysfunction. The rest of the respiratory function indexes did not change significantly when the thoracic volume became significantly reduced. We believe this is due to the lung volume not being significantly reduced, which allowed the rats to tolerate the decrease in thoracic volume.

### Changes in Chest Volume Can Assess the Severity of a Rib Fracture

Through the rat model, we elucidated the effect of reduced thoracic volume caused by isolated multiple rib fractures on respiratory function, which has a clinical application prospect. The results of this study indicate that it is feasible to evaluate the severity of multiple rib fractures by measuring the reduced thoracic volume. To some extent, this evaluation method can be used as an indication of surgical treatment.

### Limitations

This study had some limitations, the survival ability and tolerance of the rats were strong，which cannot accurately simulate the performance of human after corresponding injury in functional science. Invasive diagnostic assessment (e.g., arterial blood gas analysis) was not performed in the test rats due to ethical concerns. In addition, although the influence of external environmental factors on WBBP of experimental rats was minimized by thorough calibration before each recording, the changes of temperature, humidity, and other factors on the test day still affected the respiratory function of rats to some extent. The results of this study require further clinical validation.

### Conclusion

Displaced rib fractures lead to thoracic collapse and reduced thoracic volume, which can affect tidal volume in rats. The greater the decrease of thoracic volume, the more obvious the decrease of early postoperative tidal volume. The thoracic volume can be used as an objective index to evaluate the severity of multiple rib fractures. Early operation to restore thoracic volume may be beneficial to improve early respiratory function. Decreased thoracic volume affecting respiratory function can be compensated and recovered in long term.

## Author Contributions

Bao‐guo Jiang and Tian‐bing Wang designed this study. Jiuxu Deng and Xiao‐kun Chen performed most of the experiments and wrote the paper. Fu‐zheng Guo and Wei Huang participated in the animal surgery and acquisition of the study specimens. Feng‐xue Zhu participated in lung function experiment. Jiuxu Deng and Xiao‐kun Chen contributed to data acquisition and analysis. All authors approved the final version of the paper.

## Disclosure Statement

The authors declare that they have no competing interests.

## Authorship Declaration

All authors read and approved the final manuscript.

## Funding

National Key R&D Program of China (2018YFF0301103). UMHS‐PUHSC Joint Institute Grant (BMU 2020JI007).

## References

[1] Simon BJ , Cushman J , Barraco R , Lane V , Luchette FA , Miglietta M , et al. Pain management guidelines for blunt thoracic trauma. J Trauma. 2005;59:1256–67. 16385313 10.1097/01.ta.0000178063.77946.f5

[2] Dehghan N , de Mestral C , McKee MD , Schemitsch EH , Nathens A . Flail chest injuries: a review of outcomes and treatment practices from the National Trauma Data Bank. J Trauma Acute Care Surg. 2014;7:462–8. 10.1097/TA.0000000000000086 24458051

[3] Athanassiadi K , Theakos N , Kalantzi N , Gerazounis M . Prognostic factors in flail‐chest patients. Eur J Cardiothorac Surg. 2010;38:466–71. 20363148 10.1016/j.ejcts.2010.02.034

[4] Borman JB , Aharonson‐Daniel L , Savitsky B , Peleg K . Unilateral flail chest is seldom a lethal injury. Emerg Med J. 2006;23:903–5. 17130594 10.1136/emj.2006.037945 PMC2564248

[5] Johnston CE , McClung A , Fallatah S . Computed tomography lung volume changes after surgical treatment for early‐onset scoliosis. Spine Deform. 2014;2:460–6. 27927406 10.1016/j.jspd.2014.04.005

[6] Jaroszewski DE , Velazco CS , Pulivarthi V , Arsanjani R , Obermeyer RJ . Cardiopulmonary function in Thoracic Wall deformities: what do we really know? Eur J Pediatr Surg. 2018;28:327–46. 30103240 10.1055/s-0038-1668130

[7] DeFreest L , Tafen M , Bhakta A , Ata A , Martone S , Glotzer O , et al. Open reduction and internal fixation of rib fractures in polytrauma patients with flail chest. Am J Surg. 2016;211:761–7. 26899958 10.1016/j.amjsurg.2015.11.014

[8] Prins JTH , Van Lieshout EMM , Ali‐Osman F , et al. Outcome after surgical stabilization of rib fractures versus nonoperative treatment in patients with multiple rib fractures and moderate to severe traumatic brain injury (CWIS‐TBI). J Trauma Acute Care Surg. 2021;1(90):492–500. 10.1097/TA.0000000000002994 33093293

[9] Wijffels MME , Hagenaars T , Latifi D , Van Lieshout EMM , Verhofstad MHJ . Early results after operatively versus non‐operatively treated flail chest: a retrospective study focusing on outcome and complications. Eur J Trauma Emerg Surg. 2020;46:539–47. 29785655 10.1007/s00068-018-0961-4 PMC7280328

[10] Beks RB , de Jong MB , Houwert RM , Sweet AAR , de Bruin IGJM , Govaert GAM , et al. Long‐term follow‐up after rib fixation for flail chest and multiple rib fractures. Eur J Trauma Emerg Surg. 2019;45:645–54. 30229337 10.1007/s00068-018-1009-5 PMC6689022

[11] Beks RB , Peek J , de Jong MB , Wessem KJP , Öner CF , Hietbrink F , et al. Fixation of flail chest or multiple rib fractures: current evidence and how to proceed. A systematic review and meta‐analysis. Eur J Trauma Emerg Surg. 2019;45:631–44. 30276722 10.1007/s00068-018-1020-x PMC6689030

[12] Chien CY , Chen YH , Han ST , Blaney GN , Huang TS , Chen KF . The number of displaced rib fractures is more predictive for complications in chest trauma patients. Scand J Trauma Resusc Emerg Med. 2017;25:19. 28241883 10.1186/s13049-017-0368-y PMC5330007

[13] Chen XK , Liu YJ , Guo FZ , Deng JX , Xiong J , Wang TB , et al. Assessment of thoracic volume changes after the collapse of lateral rib fractures based on chest computed tomography data: computer simulation and a multiple variable linear regression analysis. J Cardiothorac Surg. 2020;15:167. 32646474 10.1186/s13019-020-01213-z PMC7346514

[14] Benjamin E , Recinos G , Aiolfi A , Inaba K , Demetriades D . Flail chest: less deadly than originally thought. World J Surg. 2018;42:3927–31. 29922874 10.1007/s00268-018-4723-6

[15] Flarity K , Rhodes WC , Berson AJ , Leininger BE , Reckard PE , Riley KD , et al. Guideline‐driven care improves outcomes in patients with traumatic rib fractures. Am Surg. 2017;83:1012–7. 28958283

[16] Carrie C , Guemmar Y , Cottenceau V , de Molliens L , Petit L , Sztark F , et al. Long‐term disability after blunt chest trauma: Don't miss chronic neuropathic pain! Injury. 2019;50:113–8. 30392717 10.1016/j.injury.2018.10.023

[17] Dhar SM , Breite MD , Barnes SL , Quick JA . Pulmonary contusion in mechanically ventilated subjects after severe trauma. Respir Care. 2018;63:950–4. 29535258 10.4187/respcare.05952

